# Pseudoaneurysmectomy After Left Ventricular Free Wall Rupture Repair: A Case Report

**DOI:** 10.3390/jcm14103393

**Published:** 2025-05-13

**Authors:** B. Ufuk Baldan, Patrick Klein, J. Lauran Stöger, Robert J. M. Klautz, Meindert Palmen

**Affiliations:** 1 Department of Cardiothoracic Surgery, Amsterdam University Medical Center, 1105 AZ Amsterdam, The Netherlands; 2 Department of Cardiothoracic Surgery, Leids University Medical Center, 2333 ZA Leiden, The Netherlands; 3 Department of Radiology, Leids University Medical Center, 2333 ZA Leiden, The Netherlands

**Keywords:** pseudoaneurysmectomy, left ventricular reconstruction, cardiogenic shock

## Abstract

**Background/Objectives**: Left ventricular (LV) pseudoaneurysm is a rare but life-threatening complication after acute myocardial infarction, often resulting from inadequate excision of damaged myocardium and use of only a xenopericardial patch during primary LV free wall rupture repair. **Methods**: A 62-year-old female developed a giant LV pseudoaneurysm one year after initial surgical repair of a free wall rupture with a xenopericardial patch. Imaging confirmed a large pseudoaneurysm with a broad neck and mural thrombus. She underwent pseudoaneurysmectomy, LV reconstruction with a Dacron patch overlaid by a xenopericardial patch, and concomitant mitral and tricuspid valve repair. **Results**: Surgical exploration revealed a broad-necked pseudoaneurysm and dehisced patch material. The aneurysm was resected, and the LV was reconstructed, resulting in the exclusion of the pseudoaneurysm and improvement of the shape and function. The patient recovered uneventfully and was discharged in good clinical condition with restored LV function. **Conclusions**: Pseudoaneurysm formation after LV free wall rupture repair is often due to insufficient resection and the use of only a xenopericardial patch. Surgical management with complete excision, Dacron patch reconstruction, and xenopericardial reinforcement facilitates the favorable remodeling of LV geometry and function, and reduces the risk of recurrence.

## 1. Background

True and pseudoaneurysms of the left ventricle (LV) are potentially life-threatening complications, resulting from acute transmural myocardial infarction (MI). In the acute stage of transmural MI, mechanical complications such as left LV free wall rupture, interventricular septum rupture, and papillary muscle rupture leading to acute mitral regurgitation (MR) may occur. These serious conditions necessitate immediate surgical intervention, often involving patch reconstruction in the event of a rupture. In a more post-acute phase, patients may present with an expanding true or pseudoaneurysm. When accompanied by heart failure symptoms or when cardiac function is compromised due to its location or size, surgical correction is required.

Diagnostic differentiation between these types often relies on clinical variables and medical history, as well as echocardiographic observations such as the thickness of the ventricular wall (<5 mm), an acute and sharp border between contract myocardium and aneurysm, the presence of a continuous pericardial layer, and wall motion abnormalities, such as akinesia or dyskinesia [1,2,3,4]. The incidence of both true and pseudoaneurysms is relatively low, and both entities are rarely described in literature.

## 2. Clinical Summary

**Figure 1:**
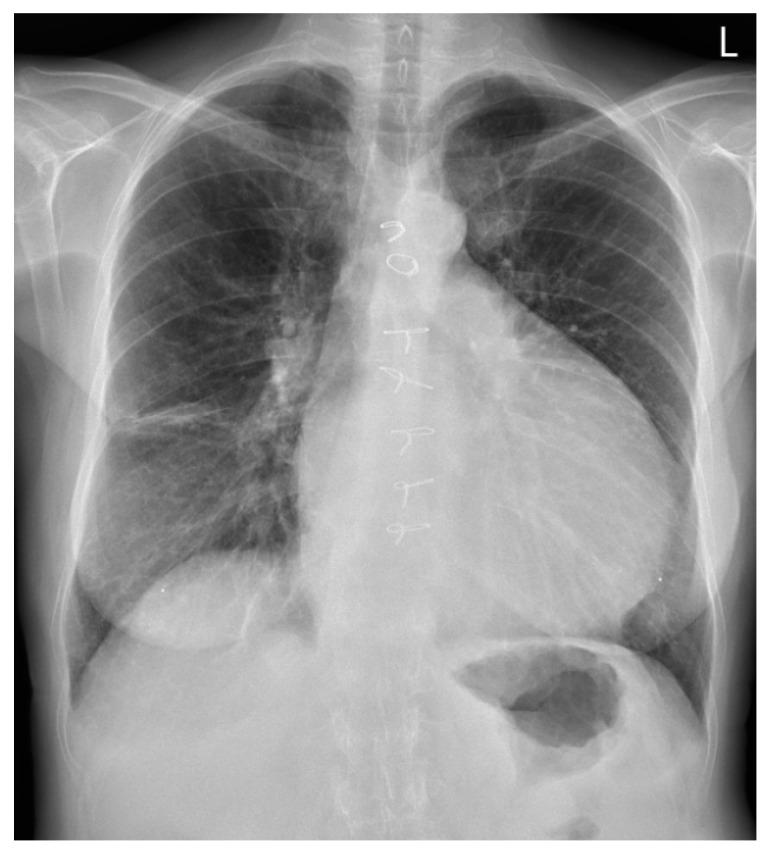
Posteroanterior chest X-ray showing marked cardiomegaly, predominantly due to the pseudoaneurysm.

A 62-year-old female patient presented in October 2021 with an MI due to occlusion of the posterolateral branch of the circumflex artery. A conservative approach was chosen at the patient’s request. The patient remained entirely asymptomatic and clinically stabile in the following month. One month later, she was re-admitted to the hospital with dyspnea and a rapid and weak pulse. Auscultation revealed signs of pulmonary edema. At that time there was clinical concern for looming cardiogenic shock; however, hemodynamic and biochemical parameters remained within normal limits. Transthoracic echocardiography (TTE) revealed an impending LV free wall rupture with pericardial tamponade, necessitating emergency surgery. Intraoperatively, a posterolateral-located LV free wall rupture was observed. The infarcted and necrotic myocardial tissue surrounding the rupture site was debrided until viable margins were achieved. The distinction between viable and non-viable myocardium is often unclear, as the demarcation of necrotic tissue may not yet be apparent. Reconstruction was accomplished by securing a bovine pericardial patch over the defect using running sutures. The patient was weaned from cardiopulmonary bypass without incident. The postoperative course was uneventful. One year later, the patient was re-evaluated due to progressive symptoms of left-sided heart failure, most notably increasing dyspnea during minimal exertion and orthopnea. She reported a marked decline in exercise tolerance, becoming noticeably more fatigued and short of breath with activities that had previously posed no difficulty, such as walking short distances or performing light household tasks. She also reported recurrent episodes of syncope. The syncopal episodes were most consistent with orthostatic hypotension and were not associated with palpitations or other features of an arrhythmogenic origin. TTE showed a dilated left atrium and ventricle with decreased function and the presence of a LV pseudoaneurysm located at the posterolateral wall. Furthermore, moderate MR and mild tricuspid regurgitation (TR) were observed. During her follow-up in 2023, she missed her biannual outpatient appointments. By the year’s end, she began experiencing increasing heart failure symptoms (New York Heart Association, class II). Routine blood tests including troponin, leukocyte count, and C-reactive protein were within normal limits. A subsequent TTE showed worsening of her MR and TR, alongside with pulmonary hypertension. More importantly, the TTE also revealed the pseudoaneurysm had increased in size, displaying a broad neck and exerting mass effect on adjacent structures. A chest X-ray, as shown in Figure 1, revealed a markedly enlarged cardiac silhouette, with a cardiothoracic ratio exceeding 0.5, and a conspicuously dilated LV contour. Additionally, Kerley B lines were observed in the right lateral lung field, accompanied by linear pleuropulmonary opacities, indicative of interstitial congestion. Consequently, she was referred from her hospital to our multidisciplinary Heart Team for high-risk patients, where she was accepted for re-operation: LV pseudoaneurysmectomy, mitral valve repair, and tricuspid valve repair.

**Figure 2:**
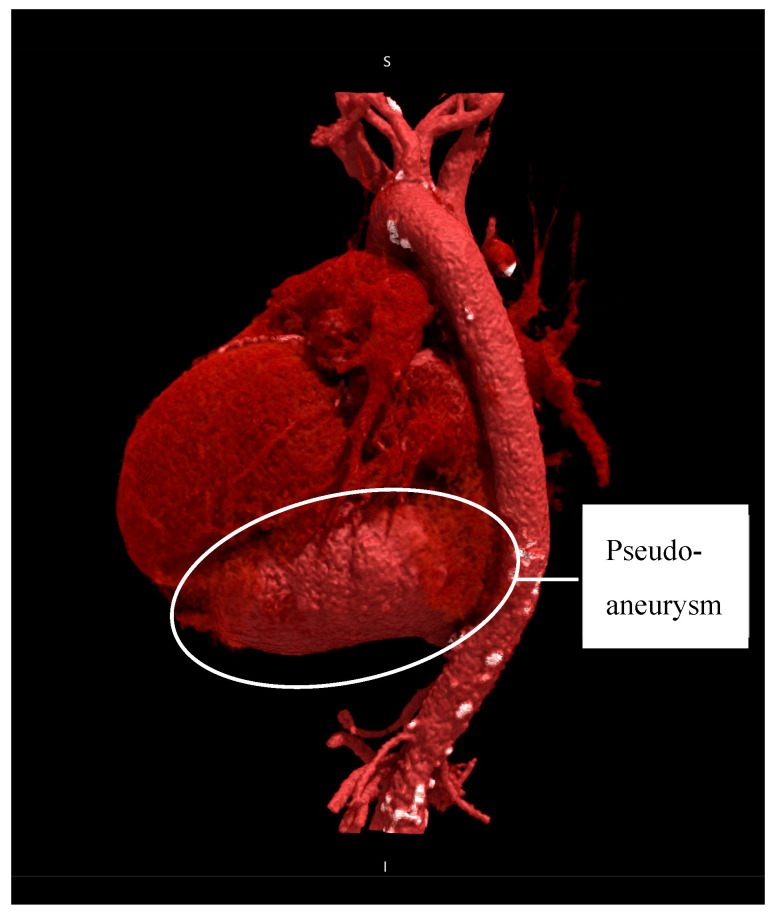
Cinematic rendering of heart and great vessels illustrating the pseudoaneurysm (12.5 × 11.6 × 8.0 cm) situated below the heart, left lateral view.

**Figure 3:**
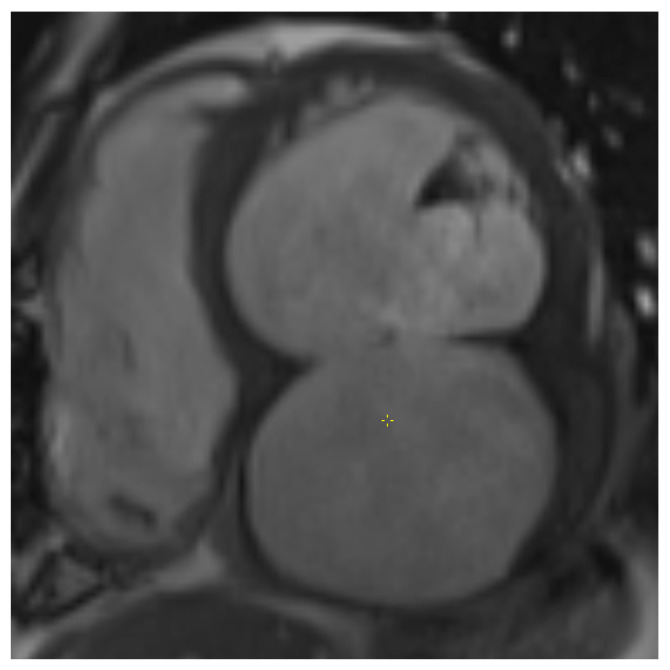
Short-axis cine image of transmural inferior wall infarction with basal inferior defect and pseudoaneurysm centered below LV and LA.

Preoperative TTE showed a moderately dilated LV with a biplane Simpson-derived ejection fraction of 31%. A giant pseudoaneurysm was identified, communicating with the true LV cavity through a broad neck, and containing a mural thrombus. No recurrent pericardial effusion was seen. Additionally, severe functional MR and TR were observed, due to systolic leaflet restriction and annular dilatation. A computed tomography angiography (CTA) illustrated an impressive LV pseudoaneurysm measuring 12.5 × 11.6 × 8.0 cm (Figure 2 and Figure 3).

**Figure 4:**
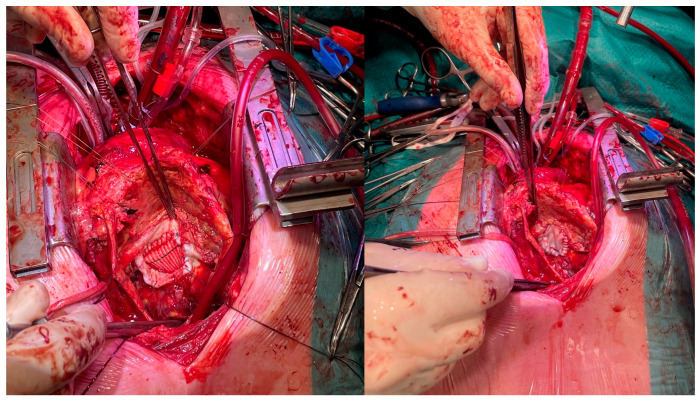
On the left side, the closure of the LV with the Dacron patch; on the right side, the Dacron patch overlayed with xenopericardial patch.

The patient was scheduled dated 18 March 2024 for urgent surgery. After median resternotomy, cardiopulmonary bypass was installed using central aortic cannulation and right femoral vein and superior caval vein canulation; the inferior vena cava was inaccessible due to the pseudoaneurysm. After dissecting the heart, the aneurysm was opened at arrested heart. After debridement and removal of the dehiscent patch material, the neck of the aneurysm in the inferior wall could be visualized and reconstructed using a Dacron patch (3 × 4 cm), which was subsequently covered with a large xenopericardial patch for hemostatic purposes, as seen in Figure 4. No histological examination of the resected pseudoaneurysm was conducted, as it was not deemed necessary for clinical decision-making. Additionally, a restrictive mitral annuloplasty using an Edwards Physio ring (size 28, downsized by 2 ring sizes) and TVP (using a size 30 Edwards Physio Tricuspid annuloplasty ring) was performed.

Following the procedure, a TTE indicated the complete exclusion of the LV pseudoaneurysm with no residual shunt. The LV shape appeared almost normal, although there was decreased systolic function, with an ejection fraction of 25%. A trace of MR (<grade I) and a mean transmitral gradient of 3 mmHg and a mild (grade I) TR were observed.

Postoperatively, the patient’s course was relatively unremarkable. She initially required vasopressors and diuretics, but she could be transferred from the ICU to the ward at postoperative day 2. She was discharged home on postoperative day 7 in good clinical condition. TTE at discharge showed no significant abnormalities, and the LV ejection fraction had largely recovered to its preoperative function (31%).

## 3. Comment

One of the most dreaded complications of a MI is an early LV free wall rupture or the formation of a true or pseudoaneurysm of the LV over time. While a free wall rupture necessitates prompt surgery, post-acute true and pseudoaneurysms of the LV can evolve differently. A pseudoaneurysm may increase in size over time and pose risks such as thrombus formation, progressing heart failure, or life-threatening arrhythmias. In rare occasions, these pseudoaneurysms can grow as large as the LV itself, necessitating surgery due to the risk of rupture. The treatment of choice involves closing the neck of the LV aneurysm using a Dacron patch, which also serves to reconstruct the LV geometry, and overlaying it with a xenopericardial patch to enhance hemostasis. In this case report, a pseudoaneurysm had formed likely due to the insufficient excision margin of the damaged myocardium and the use of a xenopericardial patch to reconstruct the LV during the primary correction of the LV free wall rupture.

The 2018 guidelines from the European Association for Cardio-Thoracic Surgery (EACTS) and the European Society of Cardiology (ESC) recommend LV aneurysmectomy primarily based on outcomes from the Surgical Treatment for Ischemic Heart Failure (STICH) trial (*n* = 49). This recommendation (class IIa, level C) emphasizes the importance of aneurysmectomy in conjunction with coronary artery bypass grafting, especially in cases involving large LV aneurysms with or without thrombus formation, and where the aneurysm poses a risk of life-threatening arrhythmias [5,6,7,8]. Notably, these guidelines do not distinguish between true and pseudoaneurysms. Given the low class and level of evidence, there is a pressing need for further research. The current literature quality is inadequate for guiding management strategies specifically for true or pseudoaneurysm formation following MI.

## 4. Conclusions

LV aneurysmectomy offers a favorable prognosis when adequate excision margins of non-viable myocardium are taken, combined with a Dacron patch to restore ventricular geometry and prevent new aneurysm formation, augmented by an overlay of xenopericardial patch to reinforce hemostasis and mitigate suture-line complications.

## Data Availability

No new data were created or analyzed in this study. Data sharing is not applicable to this article as no datasets were generated or analyzed during the current study. Further details are available from the corresponding author upon reasonable request, with respect for patient privacy and institutional regulations.
